# A Novel Small RNA-Cleaving Deoxyribozyme with a Short Binding Arm

**DOI:** 10.1038/s41598-019-44750-x

**Published:** 2019-06-03

**Authors:** Yueyao Wang, Jintao Yang, Xin Yuan, Jin Cao, Jiacui Xu, John C. Chaput, Zhe Li, Hanyang Yu

**Affiliations:** 1 Medical School of Nanjing University, Nanjing, 210093 China; 2 Department of Biomedical Engineering, College of Engineering and Applied Sciences, Nanjing University, Hankou Road, No. 22, Nanjing, 210093 China; 3 State Key Laboratory of Coordination Chemistry, School of Chemistry and Chemical Engineering, Nanjing University, Xianlin Road, No. 163, Nanjing, 210023 China; 4 College of Animal Sciences, Jilin University, Xi’an Road No. 5333, Changchun, 130062 China; 5 Department of Pharmaceutical Sciences, Department of Chemistry, and Department of Molecular Biology and Biochemistry, University of California, Irvine, California, 92697-3958 United States

**Keywords:** RNA, DNA

## Abstract

Deoxyribozymes capable of catalyzing sequence-specific RNA cleavage have found broad applications in biotechnology, DNA computing and environmental sensing. Among these, deoxyribozyme 8–17 is the most common small DNA motif capable of catalyzing RNA cleavage. However, the extent to which other DNA molecules with similar catalytic motifs exist remains elusive. Here we report a novel RNA-cleaving deoxyribozyme called 10–12opt that functions with an equally small catalytic motif and an unusually short binding arm. This deoxyribozyme contains a 14-nucleotide catalytic core that preferentially catalyzes RNA cleavage at UN dinucleotide junctions (*k*_obs_ = 0.9 h^−1^ for UU cleavage). Surprisingly, the left binding arm contains only three nucleotides and forms two canonical base pairs with the RNA substrate. Mutational analysis reveals that a riboguanosine residue 3-nucleotide downstream of cleavage site must not form canonical base pairing for the optimal catalysis, and this nucleobase likely participates in catalysis with its carbonyl O6 atom. Furthermore, we demonstrate that deoxyribozyme 10–12opt can be utilized to cleave certain microRNA sequences which are not preferentially cleaved by 8–17. Together, these results suggest that this novel RNA-cleaving deoxyribozyme forms a distinct catalytic structure than 8–17 and that sequence space may contain additional examples of DNA molecules that can cleave RNA at site-specific locations.

## Introduction

Deoxyribozymes are single-stranded DNA molecules that catalyze specific chemical reactions^1,2^. Many deoxyribozymes catalyzing a wide variety of chemical reactions have been discovered by *in vitro* selection^3–7^. Among them, DNA molecules that catalyze the sequence-specific cleavage of RNA have attracted significant attentions due to their immediate use as molecular tools in practical applications^8^. RNA-cleaving deoxyribozymes have been incorporated into technologies that impact problems ranging from biotechnology and medicine to DNA computing and metal ion sensing^9–20^. Most of these utilize the general-purpose RNA-cleaving deoxyribozymes 8–17 and 10–23 isolated by Santoro and Joyce^21^. Under the optimal conditions, 10–23 cleaves phosphodiester bonds with a catalytic efficiency of 10^9^ M^−1^min^−1^, making it the fastest nucleic acid enzyme known^21^. Deoxyribozyme 8–17, though slightly less efficient, represents an important model for RNA cleavage by a DNA sequence. This deoxyribozyme has been isolated independently from multiple *in vitro* selection experiments, suggesting that it may be one of the simplest solutions to the problem of how a DNA molecule can fold into a shape that can cleave RNA at a specific location^22–27^.

Whether DNA molecules could fold into shapes with similar catalytic activity as 8–17 but unique structural properties is an interesting question that warrants further consideration^28^. One could imagine that the simplicity of the 8–17 motif, which contains only four conserved residues in a catalytic core of 15 nucleotides, would make it relatively easy to rediscover deoxyribozymes containing such motifs by *in vitro* selection^26,29^. However, it is also possible that sequence space contains a large number of different DNA molecules that can cleave RNA. Such deoxyribozymes could be quite rare relative to the 8–17 motif or previously overlooked because they lack an obvious substrate binding site or catalytic motif^28^.

In the current work, we performed an *in vitro* selection experiment designed to sample a random pool of DNA for magnesium-dependent RNA-cleaving deoxyribozymes. We then analyzed twenty randomly picked clones for functional activity. This analysis and the following optimization allowed us to identify a deoxyribozyme called 10–12opt with an unusually short substrate-binding arm and an intriguing structural requirement outside the catalytic domain. This novel deoxyribozyme featured a 14-nucleotide catalytic core and a 3-nucleotide left binding arm. Mutational analysis confirmed that the catalytic core formed an essential 3-bp stem loop structure, and also revealed several additional essential residues near the cleavage site. Unexpectedly, a riboguanosine residue downstream of the cleavage site in RNA substrate proved critical for catalysis, and restraining this guanine nucleobase by formation of an rG:dC base pair with deoxyribozyme was detrimental to efficient catalysis. Substitution of guanine by hypoxanthine and 2-aminopurine suggested that the carbonyl O6 atom, but not the 2-amino group, of this critical G residue was necessary and sufficient to support deoxyribozyme-catalyzed RNA cleavage, likely by maintaining active catalytic structure or participating in catalysis directly. In addition to Mg^2+^, several additional divalent metal ions such as Pb^2+^, Mn^2+^, Zn^2+^ and Co^2+^ could also support the catalytic activity of deoxyribozyme 10–12opt. Lastly, we demonstrated that deoxyribozyme 10–12opt could be utilized to cleave certain microRNA sequences which are intrinsically not effectively cleaved by deoxyribozyme 8–17.

## Methods

### Materials

The DNA library was synthesized on an automated ABI 3400 DNA synthesizer, deprotected in concentrated NH_4_OH for 18 h at 55 °C, butanol precipitated, and purified by denaturing polyacrylamide gel electrophoresis (PAGE). The biotinylated DNA-RNA chimeric primer, PCR primers and deoxyribozymes were purchased from Integrated DNA Technologies (Coralville, IA) or Genscript (Nanjing, China). The fluorescently labeled RNA substrates were purchased from Takara (Dalian, China). The unmodified DNA-RNA chimeric substrate and all-RNA substrate were purchased from Dharmacon (Waltham, MA), deprotected in acetate buffer according to manufacturer’s protocol, and purified by denaturing PAGE. Klenow Fragment (exo-) DNA polymerase, T4 RNA Ligase 1, T4 polynucleotide kinase and ATP were purchased from New England BioLabs (Ipswich, MA).

### *In vitro* selection

Each round of *in vitro* selection consisted of five steps: primer extension, first strand separation, functional selection, PCR amplification and second strand separation. First, the biotinylated DNA-RNA chimeric primer was annealed to the DNA library in 1x NEBuffer 2 (50 mM NaCl, 10 mM Tris-HCl, 10 mM MgCl_2_, 1 mM DTT, pH 7.9) by heating for 5 min at 75 °C and cooling to 37 °C. Primer extension reactions contained 1 μM primer-template complex, 100 μM dNTPs, and 0.25 U/μl Klenow Fragment (exo-) DNA polymerase. Reactions were initiated by adding DNA polymerase to a solution containing all the other reagents and heating for 10 min at 37 °C. Reactions were stopped by adding an equal volume of quench buffer (2 M NaCl, 100 mM Tris-HCl, 10 mM EDTA, pH 7.0).

After primer extension, the dsDNA library was immobilized onto pre-equilibrated streptavidin-coated magnetic beads and the template strand was removed by incubation in the presence of 20 mM NaOH for 1 h at 37 °C. After extensive wash using wash buffer (1 M NaCl, 50 mM Tris-HCl, 0.1 mM EDTA, pH 7.5) to remove residual NaOH and Mg^2+^ ions, the ssDNA library with embedded ribonucleotide residues was allowed to fold and self-cleave in selection buffer (1 M NaCl, 50 mM Tris-HCl, 10 mM MgCl_2_, pH 7.5) for 3 h at 37 °C. Sequences that were able to catalyze RNA cleavage reactions were collected. To favor the enrichment of deoxyribozymes with fast catalytic rates, the incubation time was decreased incrementally from 3 h in round one to 30 min in round ten.

The recovered functional sequences were amplified by PCR using Taq DNA polymerase and PCR primers P1 and P2. The dsDNA library was made single-stranded by immobilizing the pool onto streptavidin-coated magnetic beads and eluting the template strand with 20 mM NaOH for 1 h at 37 °C. After ten rounds of *in vitro* selection and amplification, the DNA library was cloned and sequenced to examine the diversity of the molecules that remained in the pool.

### Deoxyribozyme screen

The unmodified DNA-RNA chimeric substrate was 5′-end labeled by incubation in the presence of [γ-^32^P] ATP with T4 polynucleotide kinase for 1 h at 37 °C. ^32^P-labeled substrate (500 nM) was incubated with each deoxyribozyme clone (500 nM) in screen buffer (1 M NaCl, 50 mM Tris-HCl, 50 mM MgCl_2_, pH 7.5) for 3 h at 37 °C. Reaction products were analyzed by 20% denaturing PAGE. Deoxyribozymes 8–17 and 10–23 were included as positive controls, and partial alkaline hydrolysis was performed by incubating the chimeric substrate in the presence of 200 mM NaOH for 4 min at 37 °C to generate a ladder for comparison.

### Characterization and optimization of deoxyribozyme 10–12

To evaluate the effect of single-point mutations and to probe the substrate cleavage site selectivity of the deoxyribozyme, 5′-Cy5.5-labeled RNA substrate (100 nM) and deoxyribozyme variants (100 nM) were incubated in screen buffer for 3 h at 37 °C. Following incubation, 1 μL reaction product was mixed with 20 μL ice-cold stop buffer (8 M urea, 20 mM EDTA, 5 mM Tris-HCl) and resolved on 20% denaturing PAGE. The gel was visualized and analyzed using Dual-channel Near Infrared Laser Scanning Imaging System.

### Analysis of divalent metal ion selectivity and pH dependence

Deoxyribozyme 10–12opt (100 nM) and Cy5.5-labeled RNA substrate (100 nM) were incubated under different reaction conditions. To study the effect of metal ion on deoxyribozyme catalysis, reactions were carried out with or without 1 M NaCl, in the presence of various divalent metal ions. Except for Zn^2+^ (1 mM) and Pb^2+^ (0.1 mM), the concentration of the divalent metal ion is 3 mM. The reaction pH was maintained at 7.0 except for Pb^2+^ (pH 6.0). To probe the pH dependency of deoxyribozyme-catalyzed RNA cleavage, similar reactions were carried out but at varying pH conditions. After incubating at 37 °C for 3 h, 1 μL reaction product was mixed with 20 μL ice-cold stop buffer and resolved on 20% denaturing PAGE The gel was then visualized and analyzed using Dual-channel Near Infrared Laser Scanning Imaging System.

### Kinetic measurement

Kinetic assays were carried out at 37 °C under a single turnover condition (enzyme:substrate = 100:1) with 10 nM substrate and 1 μM DNAzyme in 50 mM Tris (pH 7.5) containing 50 mM MgCl_2_, 1 M NaCl. All reactions were started at the same time. At specific time points, 1 μL reaction aliquot was removed from the water bath and quenched by mixing with 20 μL ice-cold stop buffer. The cleavage products were analyzed on denaturing PAGE.

### ATP sensor construction

The RNA substrate was conjugated to an ATP aptamer to facilitate RNA cleavage-based ATP detection. The aptamer-linked substrate (100 nM), in the absence or presence of various concentrations of ATP, was first denatured in screen buffer by heating at 70 °C for 30 s and then allowed to fold by cooling slowly to 50 °C for 50 s and holding at 4 °C. Reactions were initiated by adding deoxyribozyme 10–12opt (1 μM) and the reaction progress was monitored by removing aliquots at specific time points, mixing with ice-cold stop buffer and analyzing on denaturing PAGE.

## Results

### Identification and optimization of deoxyribozyme 10–12

**Figure 1:**
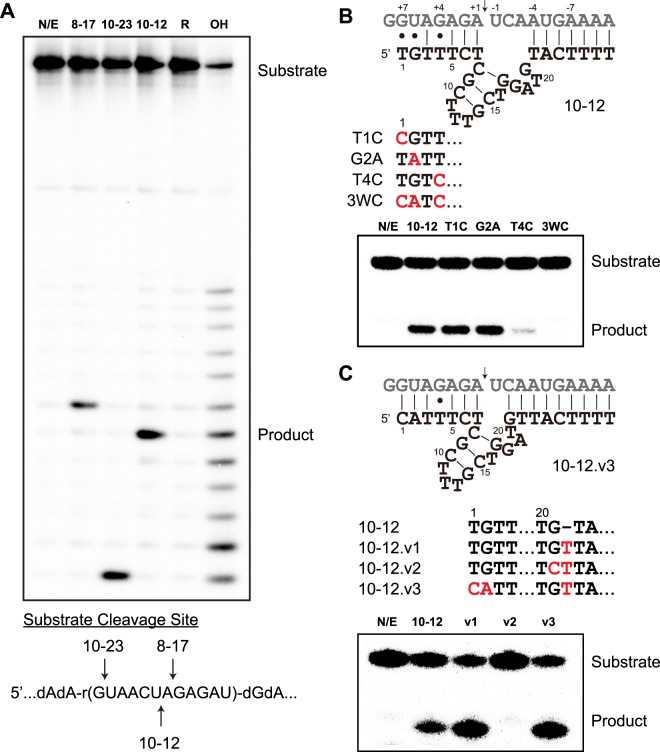
Deoxyribozyme 10–12 identification and optimization. (**A**) PAGE analysis of deoxyribozyme-catalyzed RNA cleavage revealed 10–12 cleaves substrate at a UA junction. N/E: no enzyme control. 8–17 and 10–23 are two previously reported deoxyribozymes. R: randomly generated DNA sequence. OH: partial alkaline digestion. (**B**) Single-point mutagenesis indicated that T4C mutation almost completely abolished catalytic activity. Deoxyribozyme residues are numbered from the first nucleotide in the left binding arm. The uridine and adenosine nucleotides at RNA cleavage site are named U-1 and A +1, respectively. The remaining RNA residues are numbered by counting outwards. (**C**) Optimization of 10–12 catalytic core for improved activity.

Using previously established methodology (Fig. S1), a DNA library with 50-nucleotide randomized region was subject to an RNA-cleaving catalytic activity selection^21^. Briefly, an RNA-DNA chimeric primer with 12 embedded ribonucleotides was annealed to the library template strand. The DNA library contained a central degenerate region composed of partially randomized nucleobases with a lower frequency of guanine (Table S1), which was previously used as a template strand to direct efficient synthesis of threose nucleic acid^30^. Primer extension produced a DNA library with potential RNA cleavage site attached to its 5′ end. Subsequent selection step aimed to isolate DNA molecules capable of catalyzing intramolecular Mg^2+^-dependent RNA cleavage reactions. From a starting library of 10^12^ DNA molecules, ten rounds of *in vitro* selection and amplification were performed and the representative molecules from round-10 pool were sequenced. 20 DNA molecules were synthesized and screened in trans for activity against the RNA substrate. This screen, followed by secondary structure prediction and truncation analysis, yielded a deoxyribozyme called 10–12 capable of cleaving RNA specifically at 5′-UA-3′ dinucleotide junction (Fig. 1A). Unlike many previous RNA-cleaving deoxyribozyme selection experiments, 8–17 variants were not observed in our *in vitro* selection results. 10–12 is not considered an 8–17 variant because it does not cleave DNA-RNA chimeric substrate efficiently and substituting its catalytic loop regions with 8–17 conserved residues eliminates deoxyribozyme 10–12 catalytic activity (Figs S2 and S3).

Deoxyribozyme 10–12 was predicted to form a small catalytic core of 14 nucleotides flanked with substrate-binding arms that are complementary to the RNA target (Fig. 1B). The right binding arm forms seven canonical Watson-Crick (WC) base pairs to the RNA substrate, while the left binding arm contains three putative wobble pairs at positions 1, 2, and 4 (Fig. 1B). To explore the functional significance of these non-canonical base pairs, we constructed mutant deoxyribozymes that allow for canonical WC base pairing between the deoxyribozyme and RNA substrate. When assayed for activity in a bimolecular format, deoxyribozymes with T1C and G2A mutations showed similar catalytic activities to the original enzyme, suggesting that these two putative wobble pairs are not critical for catalytic activity and could be reverted to canonical WC base pairs without compromising catalysis. However, deoxyribozymes bearing the T4C mutation either alone or in the all-Watson-Crick (3WC) construct failed to cleave the RNA substrate, implying that the putative dT:rG wobble pair at position 4 is essential for catalytic activity. We understand that it is common convention to number the nucleotides within the catalytic core and to leave out the nucleotides in the substrate-binding arms because arm length may vary in different constructs. In the current work, however, the unusual sequence requirement within the left binding arm suggested that these residues might be involved in catalysis. Therefore, to simplify the presentation of our data, we start the numbering system from the first thymidine nucleotide (T1) in the left binding and adhered to this nomenclature rule throughout the manuscript.

To explore the catalytic activity of 10–12 in greater detail, we inserted a thymidine residue after G21 to construct deoxyribozyme 10–12.v1. Such an insertion reduced the unpaired region to a single uracil residue at the scissile bond by allowing residues G21 and the newly inserted thymine to form additional base pairs with the RNA substrate (Fig. 1C). Since residue G21 was thought to form base pair with C-2 and might be important for RNA catalysis, a second deoxyribozyme 10–12.v2 was constructed that contained the mutation G21C, which forms two unpaired residues at the RNA cleavage site. A third deoxyribozyme 10–12.v3 was also constructed that reverted two non-essential wobble pairs in the left binding arm to WC base pairs. The engineered deoxyribozymes along with wild type (WT) were incubated with the RNA substrate and catalytic activities were measured by denaturing PAGE. The resulting gel indicated that deoxyribozymes carrying the thymine insertion were substantially more active than the WT enzyme. It was also evident that the guanine nucleotide at position 21 was essential for RNA cleavage as the deoxyribozyme construct bearing the G21C mutation failed to cleave the RNA substrate. Again, reverting two non-essential wobble pairs to WC base pairs had little effect on deoxyribozyme catalytic activity. Based on these results, deoxyribozyme 10–12.v3 was chosen since it exhibited the highest catalytic activity, and renamed to 10–12opt (optimized deoxyribozyme 10–12) for further study.

### Mutational analysis of deoxyribozyme 10–12opt

**Figure 2:**
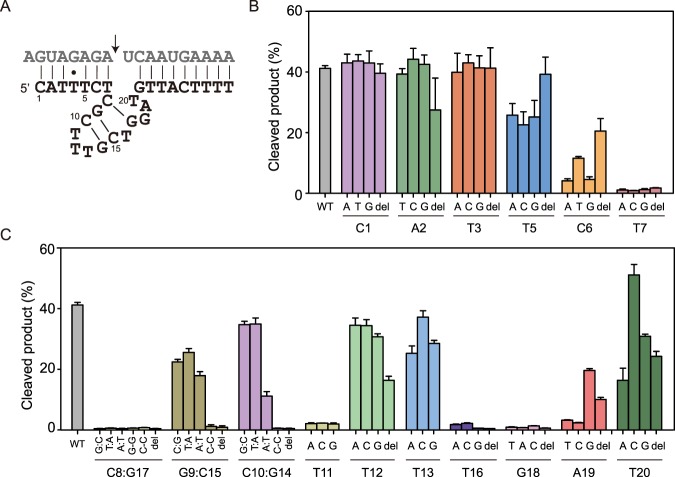
Single-point mutagenesis analysis of deoxyribozyme 10–12opt. (**A**) Predicted secondary structure of deoxyribozyme 10–12opt (black) with RNA substrate (grey). Arrowhead indicates cleavage site. (**B**) Mutational analysis of individual residues in deoxyribozyme left binding arm except T4. WT: wild type. Del: deletion. (**C**) Mutational analysis of the catalytic core region revealed several essential base pairs and residues. Deoxyribozyme mutants and RNA substrate were incubated in screen buffer for 3 h at 37 °C, followed by denaturing PAGE analysis. The error bars represent the SD calculated from three independent experiments.

The predicted secondary structure of deoxyribozyme 10–12opt resembled that of deoxyribozyme 8–17. However, it was not observed in 8–17 that formation of an rG:dC base pair within the left binding arm would result in a complete loss of catalytic activity. We thus speculated that 10–12opt was a novel RNA-cleaving deoxyribozyme with a different catalytic structure compared with 8–17. To study the functional significance of individual residues in deoxyribozyme left binding arm and catalytic core, we synthesized single-point mutants and evaluated the catalytic activity of each mutant (Fig. 2A).

A general trend regarding left binding arm mutagenesis was observed that whereas deoxyribozyme residues distant from the cleavage site were more tolerant to mutations, residues close to the cleavage site were more sensitive to substitutions. For example, although introducing mutations to the first three residues (C1, A2 and T3) in the left binding arm had little influence on catalysis, mutations to T5, C6 and T7 caused more dramatic reduction in deoxyribozyme catalytic activity (Fig. 2B). Particularly, T7 was found to be critical for catalysis, since substitution or deletion at this position abolished the deoxyribozyme activity. The catalytic core of deoxyribozyme 10–12opt consisted of three putative WC base pairs, and we introduced mutations that would disrupt, delete or maintain such base pairs. Surprisingly, it was found that C8:G17 is absolutely conserved, as disruption, deletion or even changing to other base pairs resulted in a complete loss of catalytic activity (Fig. 2C), implying that both these two nucleotides were required for catalysis and that the functional groups on these two nucleobases were probably involved in the catalytic mechanism. G9:C15 and C10:G14 were confirmed to form base pairs, since deletion or mutation to C:C abolished catalysis while changing to other canonical base pairs still retained some catalytic activities. Additional single-point mutational analysis in the catalytic core region revealed that T11, T16, G18 and A19 were conserved or semi-conserved residues, as most substitutions and deletions at these positions would result in a loss of catalytic activity (Fig. 2C).

**Figure 3:**
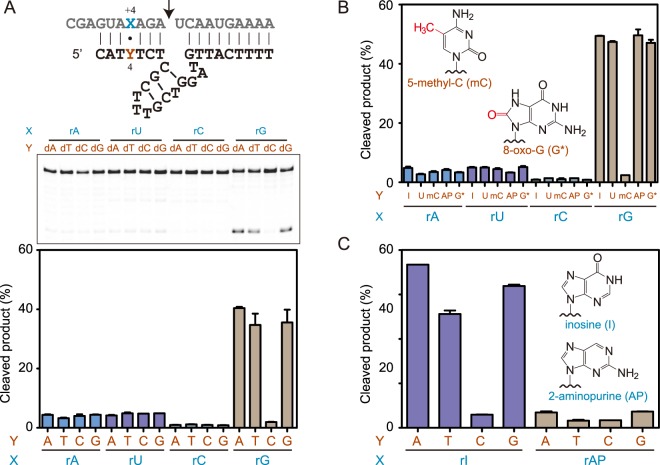
Comprehensive mutational analysis of the putative dT:rG wobble pair at position 4 of deoxyribozyme left binding arm. (**A**) PAGE analysis of 16 enzyme-substrate combinations with varied residues at position 4 of deoxyribozyme and residues at position +4 of substrate. (**B**) Substitution of residue at position 4 of deoxyribozyme with inosine (I), uridine (U), 5-methylcytidine (mC), 2-aminopurine (AP) and 8-oxoguanosine (G*). (**C**) Substitution of the critical guanosine residue of RNA substrate with inosine and 2-aminopurine analogues. Variants of deoxyribozyme and RNA substrate were incubated in screen buffer for 3 h at 37 °C, followed by denaturing PAGE analysis. The error bars represent the SD calculated from three independent experiments.

To determine whether a putative dT:rG wobble pair at position 4 is necessary for catalysis, we constructed four deoxyribozymes and four substrates with varied nucleotide identities at this putative wobble pair position. When these 16 enzyme-substrate combinations were assayed for RNA cleavage, it was discovered unexpectedly that significant cleavage was only observed when RNA substrate contains a guanine at the +4 position and on the opposite position of deoxyribozyme is not cytosine (Fig. 3A), suggesting that the guanine residue on substrate played a critical structural and/or catalytic role and that forming an rG:dC base pair at this position would restrain and interfere with guanine’s function and result in a complete loss of catalytic activity.

It has not been observed in any previously reported RNA-cleaving deoxyribozymes that a riboguanosine residue downstream of the cleavage site was critical for catalysis. It was even more fascinating that formation of canonical rG:dC base pair at this position completely abolished deoxyribozyme catalytic activity. Intrigued by this observation, we employed modified nucleobase analogue substitutions to investigate the role of nucleobase functional group. We first introduced base mutations to position 4 of deoxyribozyme, using hypoxanthine, 2-aminopurine and 8-oxoguanine as purine analogues and using uracil and 5-methylcytosine as pyrimidine analogues. Consistent with earlier natural base substitution results, RNA substrates with A, U and C residues at the +4 position were not efficiently cleaved, no matter what nucleobase analogue was used at position 4 of the deoxyribozyme (Fig. 3B). Only RNA substrate with guanine at this position was efficiently cleaved, as long as the deoxyribozyme contained a nucleobase other than cytosine or its analogue at position 4 (Fig. 3B). To dissect the functional roles of the carbonyl and amino groups of this guanine residue, we generated RNA substrates with G +4 replaced by hypoxanthine and 2-aminopurine, respectively. Cleavage assays using such RNA substrates and four deoxyribozymes revealed that only the inosine substrate variant was efficiently cleaved and maintained the sequence preference against cytosine at position 4 of deoxyribozyme. Substitution of guanine with 2-aminopurine rendered the RNA substrate not efficiently cleaved by any of the four deoxyribozymes (Fig. 3C). All these results indicated that the riboguanosine residue located 3-nucleotide downstream of the cleavage site played a critical role in deoxyribozyme-catalyzed RNA cleavage, probably via a direct participation in catalysis of its O6 atom, but not the exocyclic amino group.

**Figure 4:**
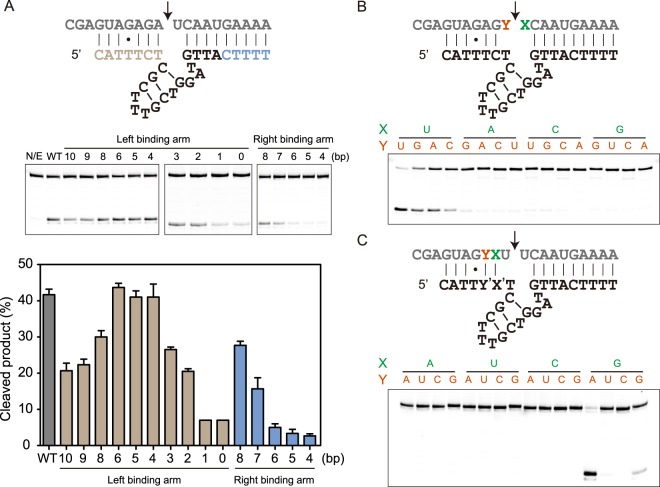
Effect of binding arm length on catalysis and substrate sequence preference. (**A**) Effect of different lengths of left and right binding arms on RNA cleavage activity. Cleavage reactions catalyzed by different deoxyribozyme constructs were assayed on three separate gels and gel images were grouped together for comparison. (**B**) PAGE analysis of deoxyribozyme-catalyzed cleavage of RNA substrates containing 16 different cleavage site dinucleotide junctions. (**C**) PAGE analysis of deoxyribozyme-catalyzed cleavage of RNA substrates containing 16 different combinations of nucleotides at +2 and +3 positions. RNA substrates and deoxyribozyme variants with compensatory mutations were incubated in screen buffer for 3 h at 37 °C, followed by denaturing PAGE analysis. The error bars represent the SD calculated from three independent experiments.

Having observed that the first three residues of deoxyribozyme left binding could be mutated without affecting its catalytic activity, and that the fourth position had a strong and unusual preference against cytosine, we wondered what the impacts of different substrate-binding arm lengths on deoxyribozyme catalytic activity would be. Therefore we synthesized deoxyribozyme constructs of various binding arm lengths. As expected, the catalytic activity of deoxyribozyme gradually decreased with incrementally shortened right binding arm, presumably due to weakened binding affinity to the substrate. However, when the length of left binding arm was varied, the deoxyribozyme exhibited maximal catalytic activity when the arm length was between four and seven base pairs (Fig. 4A). Further increasing the left binding arm length beyond 7 bp unexpectedly caused a reduction in catalytic efficiency. This unusual observation, together with the strict requirement that an rG:dC base pair must not form at the fourth position, implied that formation of a perfect duplex downstream of the cleavage site was deleterious for efficient catalysis. It seemed necessary for the rG +4 residue to have some flexibility to endow the deoxyribozyme with optimal catalytic activity. Since the first three nucleotides of deoxyribozyme 10–12opt could be deleted without affecting catalysis, we synthesized a shortened version of deoxyribozyme 10–12opt with the first three residues deleted, and sought to determine whether it still maintained the unusual dD:rG (D = A, T, G) sequence requirement. Indeed, only RNA substrates with guanine nucleotide at the +4 position could be cleaved by the shortened deoxyribozyme variants, and the shortened deoxyribozyme with cytosine at position 4 also showed reduced catalytic activity (Fig. S4). The observed reduction in catalytic efficiency was modest in the shortened deoxyribozyme compared to the original 10–12opt. The possible reason might be that the formation of rG:dC base pair in the middle of a duplex reduced the flexibility of rG +4 required for optimal catalysis, whereas in the shortened deoxyribozyme the rG:dC base pair was terminal and not as stable, more likely to undergo spontaneous breathing and not restraining the rG residue as tightly. We also evaluated catalytic activities of deoxyribozyme variants with simultaneously shortened left and right binding arms (Fig. S5). As expected, the deoxyribozyme catalytic activity generally decreased as the binding arm lengths were shortened simultaneously.

We studied the RNA cleavage site selectivity of deoxyribozyme 10–12opt. When assayed against RNA substrates with 16 different cleavage site dinucleotide junctions, 10–12opt exhibited preferential cleavage activities, with 5′-UN-3′ as the most favorable cleavage junction in the order of UU > UG > UA > UC. As shown in Fig. 4B, deoxyribozyme 10–12opt did not appreciably cleave RNA substrates with the other 12 dinucleotide junctions. This substrate preference was particularly intriguing, considering that thymine at position 7 on deoxyribozyme had been proved essential for efficient catalysis (Fig. 2B). Initially it was assumed that it contributed to scissile bond positioning by forming a canonical base pair with the rA +1 nucleotide on RNA substrate (Fig. 2A). However, the cleavage site selectivity assay revealed that deoxyribozyme 10–12opt preferentially cleaved RNA substrates with UN, but not NA, dinucleotide junctions (Fig. 4B), implying that a canonical WC base pair at this position was not required. A systematic mutagenesis analysis at this particular location confirmed that thymine at position 7 on deoxyribozyme was absolutely conserved for efficient catalysis and that formation of a WC base pair at this position was neither required nor preferred for optimal RNA cleavage (Fig. S6). The mutagenesis analysis on the shortened deoxyribozyme 10–12opt revealed a similar sequence requirement for T7 and a modest preference for non-cytosine residue at position 4 (Fig. S7).

Considering the deoxyribozyme showed strong substrate sequence requirement and preference at positions −1, +1 and +4 (Figs 3A, 4B), we sought to explore whether nucleotide mutations occurring at positions +2 and +3 were tolerated. We synthesized all the other RNA substrate variants bearing one of the 16 possible combinations at these two positions, and examined their cleavage results by the corresponding deoxyribozymes with compensatory mutations. Surprisingly, only the original substrate (G +2 and A +3) and one other variant (G +2 and G +3) were effectively cleaved, with the former being the preferred substrate sequence. All the remaining 14 RNA sequences did not yield appreciable cleavage products, even though compensatory mutations were introduced to deoxyribozyme sequence to maintain the Watson-Crick base pairing at these two positions. Based on the results from the mutational analysis, the deoxyribozyme 10–12opt selectively cleaves RNA substrates with UNGRG consensus sequence (N = U > G > A > C and R = A > G).

### Metal ion selectivity, pH dependence and reaction kinetics

**Figure 5:**
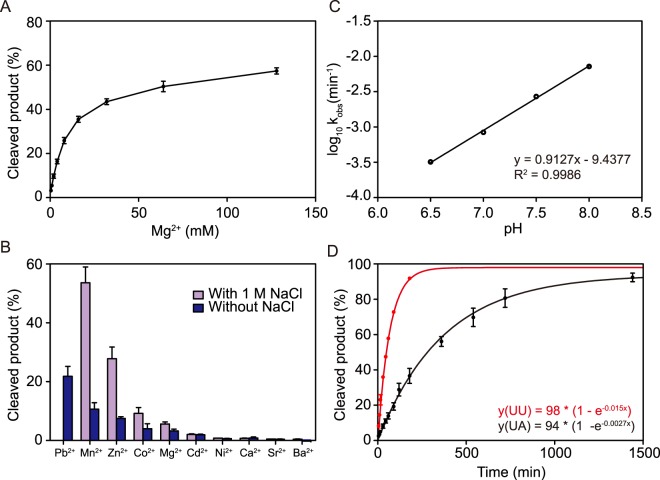
Characterization of deoxyribozyme 10–12opt. (**A**) Deoxyribozyme catalytic activity depends on magnesium ion. (**B**) Metal ion selectivity. Metal ion concentration is 3 mM except for Zn^2+^ (1 mM) and Pb^2+^ (0.1 mM). (**C**) pH dependence of the cleavage reaction rate. The data was fitted linearly with a slope of 0.91. (**D**) Kinetics of deoxyribozyme 10–12opt catalyzed RNA cleavage reactions occurring at UU and UA junctions. Kinetics experiments were performed under a single turnover condition (enzyme:substrate = 100:1) in 50 mM Tris buffer solution (pH 7.5) containing 50 mM MgCl_2_, 1 M NaCl. The error bars represent the SD calculated from three independent experiments.

The deoxyribozyme 10–12 was originally selected in the presence of 10 mM Mg^2+^, and as expected the catalytic activity of 10–12opt increases in a magnesium-dependent manner (Fig. 5A). To determine the metal ion dependence of deoxyribozyme, we assayed the deoxyribozyme catalytic activity in the presence of various divalent metal ions (Fig. 5B). Similar to previously reported RNA-cleaving deoxyribozymes, several other metal ions supported the catalytic activity of deoxyribozyme 10–12opt in the presence of NaCl in the order of Mn > Zn > Co > Mg > Cd. Pb^2+^ was omitted because it resulted in PbCl_2_ precipitation under our reaction conditions. The catalytic activity of 10–12opt was also examined in the absence of NaCl. Interestingly, when NaCl is not present in the reaction, several divalent metal ions were still able to enhance the deoxyribozyme-catalyzed RNA cleavage, but to a less extent, following a similar order of Pb > Mn > Zn > Co > Mg > Cd. This trend is roughly the reverse order of the pK_a_ values of the corresponding metal hydrates, implying that a coordinated metal hydroxide might act as a general base and deprotonate the 2′-hydroxyl at the cleavage site. This correlation between catalytic activity and pK_a_ value does not hold for all the metal ions and shows some exceptions, which have also been observed in other ribozymes and deoxyribozymes. While Mg^2+^ supported deoxyribozyme activity similarly in the presence and absence of NaCl (Fig. 5B), Mn^2+^ could significantly enhance 10–12opt catalyzed RNA cleavage in the presence of 1 M NaCl compared with deoxyribozyme performance in the absence of NaCl (Fig. S8).

To probe the deoxyribozyme catalytic mechanism, we determined the effect of reaction pH on the deoxyribozyme catalytic efficiency, and identified the RNA cleavage products. It was discovered that the log value of observed reaction rate increased linearly with a slope of 0.9 as pH increased (Fig. 5C), implying the reaction proceeds via a classical mechanism involving a single deprotonation event of ribose 2′-OH followed by nucleophilic attack on the phosphorus atom. Consistent with this mechanism, the cleavage products were identified to be a 2′,3′-cyclic phosphate and a free 5′ hydroxyl group (Fig. S9)^21,24,31,32^.

To quantify the catalytic activity of 10–12opt, we measured the reaction rates of deoxyribozyme-catalyzed RNA cleavage under single turnover conditions. Briefly, 10 nM RNA substrate was incubated with 1 μM deoxyribozyme (substrate/enzyme ratio = 1:100) in 50 mM Tris (pH 7.5) buffer containing 50 mM MgCl_2_ and 1 M NaCl. At specific time points, reaction aliquots were removed and analyzed on denaturing PAGE. The cleaved product percentage and time were used to determine the observed rate using the equation: Y = Ymax (1 − e^(-kt)) where Ymax was the yield of a 24-hour reaction. As illustrated in Fig. 5D, 10–12opt exhibits a catalytic rate (*k*_obs_) of 1.5 × 10^−2^ min^−1^ and 2.7 × 10^−3^ min^−1^ towards RNA substrates with UU and UA cleavage site junctions, respectively.

### Detection system for microRNA and ATP

**Figure 6:**
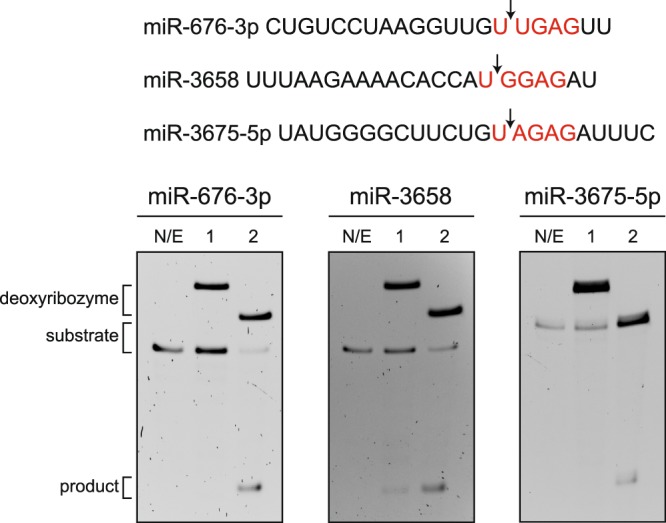
Deoxyribozyme 10–12opt catalyzed microRNA cleavage. Three microRNA sequences containing UNGRG consensus sequence were each treated with deoxyribozyme 8–17 and 10–12opt respectively. While 8–17 was able to cleave only microRNA-3658 but not the other two, deoxyribozyme 10–12opt catalyzed site-specific cleavage of each microRNA substrate. N/E: no enzyme control. Lane 1: microRNA treated with deoxyribozyme 8–17. Lane 2: microRNA treated with deoxyribozyme 10–12opt.

Given that deoxyribozyme 10–12opt features an unusually short left binding arm and selectively cleaves RNA substrates containing UNGRG consensus sequence, we reasoned that such deoxyribozyme could be used to cleave microRNA (miR), which is intrinsically short in length and plays diverse regulatory roles in biology. We searched the human microRNA database for microRNA sequences that meet the above requirements, and chose miR-676-3p, miR-3658 and miR-3675-5p as substrates because they each contain UU, UG and UA cleavage site junctions, respectively. For comparison, deoxyribozyme 8–17 was included because it exhibited low catalytic activity at such cleavage sites. As expected, deoxyribozyme 10–12opt could effectively cleave all three microRNA substrates (Fig. 6). On the other hand, deoxyribozyme 8–17 could only cleave microRNA-3658, which contain UG dinucleotide junction at the cleavage site, but could not catalyze cleavage reactions of the other two RNA substrates, which contain UU and UA cleavage site, respectively, reflecting the inherent low activity of 8–17 towards such cleavage sites. These results demonstrated that deoxyribozyme 10–12opt could be used to catalyzed site-specific cleavage reactions of short RNA sequences that are not efficiently cleaved by commonly used 8–17 deoxyribozyme.

To demonstrate that deoxyribozyme 10–12opt could be used together with an aptamer to generate an analyte-responsive RNA-cleaving enzyme, we conjugated the RNA substrate to an ATP-binding DNA aptamer to build a molecular ATP detection platform (Fig. S10A)^33–35^. When ATP was not present in the system, the DNA-RNA chimeric molecule formed an intramolecular hairpin structure that precluded binding of deoxyribozyme. When ATP was present in the system, however, binding-induced structural change exposed the RNA substrate, making it accessible to the deoxyribozyme and susceptible to cleavage. Therefore, in the presence of ATP, the designed system yielded specific cleavage product whereas the same construct remained intact in the absence of ATP (Fig. S10B). Time course experiments with varying concentration of ATP as well as other potentially interfering NTPs clearly demonstrated that the detection system could selectively respond to ATP and generate cleavage product in a concentration- and time-dependent fashion (Fig. S10C).

## Discussion

Here, we reported a novel small RNA-cleaving deoxyribozyme 10–12opt with the shortest binding arm and an unusual sequence requirement within this region. Although the predicted secondary structure of deoxyribozyme 10–12opt looked initially similar to that of deoxyribozyme 8–17, careful examination of the sequence requirement clearly revealed that 10–12opt was a novel RNA-cleaving deoxyribozyme, but not an 8–17 variant. First, there was an unusual sequence requirement of both deoxyribozyme 10–12opt and RNA substrate. Located 3-nucleotide downstream of the cleavage site, the riboguanosine residue proved critical for deoxyribozyme-catalyzed RNA cleavage. This nucleobase possibly participated directly in catalysis or maintained the integrity of the catalytic structure, with its carbonyl O6 atom likely acting as a hydrogen bond acceptor. Indeed, when this guanine was replaced by other nucleobases (adenine, cytosine, thymine and 2-aminopurine), or when this guanine was restrained in a perfect duplex by formation of a canonical rG:dC base pair, the catalytic activity of the deoxyribozyme was greatly diminished. This has not been observed in 8–17 or any reported RNA-cleaving deoxyribozymes. Furthermore, deoxyribozyme 8–17 has four conserved residues (A5, G6, C12 and G13) with tertiary interactions within its catalytic core^36^. Substituting the TTT and GAT loops within deoxyribozyme 10–12opt catalytic core with the corresponding sequences found in 8–17 (AGC and ACGA, respectively) rendered deoxyribozyme catalytically inactive (Fig. S3). Lastly, while 8–17 catalyzes RNA cleavage reactions of both all-RNA substrate and DNA substrate with a single ribonucleotide embedded at cleavage site, deoxyribozyme 10–12opt only efficiently cleaved all-RNA substrate but showed a minimal catalytic activity towards a DNA-RNA chimeric substrate (Fig. S2). All these results suggested that 10–12opt was a novel RNA-cleaving deoxyribozyme with a distinct catalytic conformation.

Almost all RNA-cleaving deoxyribozymes discovered thus far feature two substrate-binding arms and a central catalytic core. Initially, deoxyribozyme 10–12opt appeared to adopt a similar structure. However, careful mutagenesis analysis suggested that it might adopt a different catalytic conformation. It was found that substitution to or deletion of the first three nucleotides (C1, A2 and T3) of the 7-bp left binding arm had little influence on deoxyribozyme catalytic activity (Figs 2, 4), suggesting that these residues are dispensable for catalysis. On the other hand, mutating the last three nucleotides (T5, C6 and T7) caused significant or modest reduction in catalytic activity (Figs 2, 4), indicating that these residues are required for optimal catalysis. The critical role thymine at position 7 played in catalysis was particularly intriguing. It was initially perceived to contribute to the scissile bond positioning by forming a dT:rA base pair with the substrate. However, a following cleavage site selectivity experiment proved otherwise. Deoxyribozyme catalyzed RNA cleavage preferentially at UN, but not NA junctions. Among the substrates with UN junctions, UA substrate was not the most favorable but it followed an order of UU > UG > UA > UC, implying that a canonical base pair at this position was not required for optimal catalysis. Indeed, it was later confirmed by a systematic analysis that thymine itself, but not formation of a base pair, at position 7 was required for efficient catalysis (Fig. S6). A similar observation was previously made by Silverman and co-workers in their deoxyribozymes that synthesize 2′,5′-branched RNA^37^. A uracil residue near the ligation site was found to be critical for catalysis, and initially suspected to form a base pair with the deoxyribozyme nucleotide G or A. However, substitution and compensatory mutational analysis indicated that this residue was not interacting with deoxyribozyme via a simple base pairing.

Since the first three nucleotides could be deleted without compromising catalytic activity and T7 was not involved in base pairing with the RNA residue at +1 position, we reasoned that deoxyribozyme 10–12opt contained a 3-nucleotide left binding arm and 14-nucleotide catalytic core. Interestingly, only two nucleotides (T5 and C6) formed canonical base pairing with the RNA substrate, and the residue at position 4 must not form WC base pairing with G +4 on the RNA substrate for optimal catalysis. We speculated that the riboguanosine nucleotide on RNA substrate played a critical role in maintaining the deoxyribozyme catalytic structure or participated directly in the catalytic mechanism using its carbonyl O6 atom as a potential hydrogen bond acceptor. In fact, RNA substrates with non-guanosine nucleotides at +4 position were not efficiently cleaved by any 10–12opt variants. Moreover, deoxyribozyme mutant with cytosine at position 4, which formed a canonical dC:rG base pair, exhibited little or reduced catalytic activity, implying that at least some flexibility of G +4 was necessary for optimal catalysis and restraining this essential guanine in a perfect duplex conformation lead to significantly reduced catalytic activity. To the best of our knowledge, this RNA-cleaving deoxyribozyme holds the shortest binding arm thus far, and the RNA substrate is the first one with an essential guanine residue away from the cleavage site, which must not form WC base pairing with deoxyribozyme. While not entirely identical to our shortened deoxyribozyme 10–12opt, one of Breaker’s oxidative DNA-cleaving deoxyribozymes also has an unusual and rather short triplex binding arm structure^38^. Altogether, these results suggested that the role of the unusually short left binding arm was not limited to substrate binding. Instead, the requirement that a canonical base pair must not form at position 4 indicated that that at least the riboguanosine nucleotide was directly involved in catalysis. Since our deoxyribozyme 10–12opt features an unusually short substrate-binding arm, it could be potentially useful in assays where RNA substrate is short and near-terminus site-specific cleavage is desired. Moreover, 10–12opt cleaves all-RNA substrates preferentially at UN dinucleotide junctions, and thus could be practically complementary to the previously reported 8–17 and 10–23 deoxyribozymes.

Deoxyribozyme 10–12opt provides an interesting example of a DNA molecule that catalyzes RNA cleavage at a site-specific location, which represents a step forward in the discovery of diverse DNA architectures that cleave RNA. To our knowledge, 10–12opt is the first example of an RNA-cleaving deoxyribozyme that contains only three nucleotides as its left binding arm. Within this region, a guanine on RNA substrate proves critical for catalytic activity and it must not form a canonical rG:dC base pair with deoxyribozyme for optimal catalysis. All these unusual sequence requirements and preferences suggest that 10–12opt forms a discrete catalytic architecture, different from all known RNA-cleaving DNA enzymes. This discovery provides the motivation to search combinatorial libraries for DNA molecules with even more diverse structural features. Obtaining an unbiased view of the fitness landscape should be possible with recent advances in next-generation deep sequencing technologies, which allow for a large sampling of DNA sequences^39,40^. As these experiments progress, it will be interesting to learn how deoxyribozymes are distributed in sequence space and how well sequence copy number correlates with functional activity.

## Conclusion

We have identified a novel small RNA-cleaving deoxyribozyme with an unusually short (3-nucleotide) left substrate-binding arm, which preferentially catalyzes RNA cleavage at UN junctions. A riboguanosine nucleotide within the left binding arm proves critical for catalysis, and its O6 atom is likely involved in catalysis. Taking advantage of its short left binding arm, it was further demonstrated that this deoxyribozyme could be used to catalyze site-specific cleavage of short microRNA sequences containing the UNGRG consensus sequence near the 3′ end. This deoxyribozyme represents a unique catalytic DNA structure capable of promoting site-specific RNA cleavage.

## Supplementary information

supplementary info

## Data Availability

All data generated or analyzed during this study are included in this published article (and its Supplementary Information files).

## References

[1] Breaker RR, Joyce GF (1994). A DNA enzyme that cleaves RNA. Chem. Biol..

[2] Breaker RR, Joyce GF (1995). A DNA enzyme with Mg^2+^-dependent RNA phosphoesterase activity. Chem. Biol..

[3] Li Y, Breaker RR (1999). Deoxyribozymes: new players in the ancient game of biocatalysis. Curr. Opin. Struct. Biol..

[4] Emilsson GM, Breaker RR (2002). Deoxyribozymes: new activities and new applications. Cell. Mol. Life Sci..

[5] Peracchi A (2005). DNA catalysis: potential, limitations, open questions. Chembiochem.

[6] Silverman, S. K. Catalytic DNA (deoxyribozymes) for synthetic applications-current abilities and future prospects. *Chem. Commun*., 3467–3485 (2008). 10.1039/b807292m 18654692

[7] Silverman SK (2009). Deoxyribozymes: selection design and serendipity in the development of DNA catalysts. Acc. Chem. Res..

[8] Silverman SK (2005). *In vitro* selection, characterization, and application of deoxyribozymes that cleave RNA. Nucleic Acids Res.

[9] Achenbach JC, Chiuman W, Cruz RP, Li Y (2004). DNAzymes: from creation *in vitro* to application *in vivo*. Curr. Pharm. Biotechnol..

[10] Lu Y, Liu J (2006). Functional DNA nanotechnology: emerging applications of DNAzymes and aptamers. Curr. Opin. Biotechnol..

[11] Baum DA, Silverman SK (2008). Deoxyribozymes: useful DNA catalysts *in vitro* and *in vivo*. Cell. Mol. Life Sci..

[12] Willner I, Shlyahovsky B, Zayats M, Willner B (2008). DNAzymes for sensing, nanobiotechnology and logic gate applications. Chem. Soc. Rev..

[13] Ali MM, Aguirre SD, Lazim H, Li Y (2011). Fluorogenic DNAzyme probes as bacterial indicators. Angew. Chem. Int. Ed..

[14] Wu P, Hwang K, Lan T, Lu Y (2013). A DNAzyme-gold nanoparticle probe for uranyl ion in living cells. J. Am. Chem. Soc..

[15] McGhee CE, Loh KY, Lu Y (2017). DNAzyme sensors for detection of metal ions in the environment and imaging them in living cells. Curr. Opin. Biotechnol..

[16] Wu Z (2017). Imaging Endogenous Metal Ions in Living Cells Using a DNAzyme-Catalytic Hairpin Assembly Probe. Angew. Chem. Int. Ed..

[17] Liu M (2017). A DNAzyme Feedback Amplification Strategy for Biosensing. Angew. Chem. Int. Ed..

[18] Torabi SF (2015). *In vitro* selection of a sodium-specific DNAzyme and its application in intracellular sensing. Proc. Natl. Acad. Sci. USA.

[19] Zhou W, Zhang Y, Huang PJ, Ding J, Liu J (2016). A DNAzyme requiring two different metal ions at two distinct sites. Nucleic Acids Res..

[20] Zhou W, Saran R, Huang PJ, Ding J, Liu J (2017). An Exceptionally Selective DNA Cooperatively Binding Two Ca(2+) Ions. Chembiochem.

[21] Santoro SW, Joyce GF (1997). A general purpose RNA-cleaving DNA enzyme. Proc. Natl. Acad. Sci. USA.

[22] Faulhammer D, Famulok M (1996). The Ca^2+^ ion as a cofactor for a novel RNA-cleaving deoxyribozyme. Angew. Chem. Int. Ed..

[23] Peracchi A (2000). Preferential activation of the 8–17 deoxyribozyme by Ca^2+^ ions - Evidence for the identity of 8–17 with the catalytic domain of the MG5 deoxyribozyme. J. Biol. Chem..

[24] Li J, Zheng WC, Kwon AH, Lu Y (2000). *In vitro* selection and characterization of a highly efficient Zn(II)-dependent RNA-cleaving deoxyribozyme. Nucleic Acids Res..

[25] Schlosser K, Li YF (2004). Tracing sequence diversity change of RNA-cleaving deoxyribozymes under increasing selection pressure during *in vitro* selection. Biochemistry.

[26] Cruz RPG, Withers JB, Li YF (2004). Dinucleotide junction cleavage versatility of 8–17 deoxyribozyme. Chem. Biol..

[27] Schlosser K, Gu J, Lam JCF, Li YF (2008). *In vitro* selection of small RNA-cleaving deoxyribozymes that cleave pyrimidine-pyrimidine junctions. Nucleic Acids Res..

[28] Lam JCF, Kwan SO, Li YF (2011). Characterization of non-8–17 sequences uncovers structurally diverse RNA-cleaving deoxyribozymes. Mol. Biosyst..

[29] Schlosser K, Li YF (2010). A Versatile Endoribonuclease Mimic Made of DNA: Characteristics and Applications of the 8–17 RNA-Cleaving DNAzyme. Chembiochem.

[30] Yu H, Zhang S, Chaput JC (2012). Darwinian evolution of an alternative genetic system provides support for TNA as an RNA progenitor. Nat. Chem..

[31] Brown AK, Li J, Pavot CMB, Lu Y (2003). A lead-dependent DNAzyme with a two-step mechanism. Biochemistry.

[32] Schlosser K, Li YF (2009). DNAzyme-mediated catalysis with only guanosine and cytidine nucleotides. Nucleic Acids Res..

[33] Huizenga DE, Szostak JWA (1995). DNA Aptamer That Binds Adenosine and ATP. Biochemistry.

[34] Chiuman W, Li YF (2007). Simple Fluorescent Sensors Engineered with Catalytic DNA ‘MgZ’ Based on a Non-Classic Allosteric Design. PLoS One.

[35] Tram K, Xia JJ, Gysbers R, Li YF (2015). An Efficient Catalytic DNA that Cleaves L-RNA. PLoS One.

[36] Liu HH (2017). Crystal structure of an RNA-cleaving DNAzyme. Nat. Commun..

[37] Wang YM, Silverman SK (2003). Characterization of deoxyribozymes that synthesize branched RNA. Biochemistry.

[38] Carmi N, Breaker RR (2001). Characterization of a DNA-cleaving deoxyribozyme. Biorg. Med. Chem..

[39] Ameta S, Winz ML, Previti C, Jaschke A (2014). Next-generation sequencing reveals how RNA catalysts evolve from random space. Nucleic Acids Res..

[40] Pressman A, Moretti JE, Campbell GW, Muller UF, Chen IA (2017). Analysis of *in vitro* evolution reveals the underlying distribution of catalytic activity among random sequences. Nucleic Acids Res..

