# Supplementary material for: A Novel Small RNA-Cleaving Deoxyribozyme with a Short Binding Arm
Source: Sci Rep. 2019 Jun 3;9:8224. doi: 10.1038/s41598-019-44750-x (PMC6546695; doi:10.1038/s41598-019-44750-x)
Supplement: Supplementary file 1 — supplementary info [file 41598_2019_44750_MOESM1_ESM.docx]

**Supplementary Information**

**A Novel Small RNA-Cleaving Deoxyribozyme with a Short Binding Arm**

Yueyao Wang^1^, Jintao Yang^2^, Xin Yuan^3^, Jin Cao^2^, Jiacui Xu^4^, John Chaput^5^, Zhe Li*^2^, and Hanyang Yu*^2^

^1^Medical School of Nanjing University, ^2^Department of Biomedical Engineering, College of Engineering and Applied Sciences, Nanjing University, Hankou Road, No. 22, Nanjing 210093, China.

^3^State Key Laboratory of Coordination Chemistry, School of Chemistry and Chemical Engineering, Nanjing University, Xianlin Road, No. 163, Nanjing 210023, China.

^4^College of Animal Sciences, Jilin University, Xi’an Road No. 5333, Changchun 130062, China.

^5^Department of Pharmaceutical Sciences, Department of Chemistry, and Department of Molecular Biology and Biochemistry, University of California, Irvine, California 92697-3958, United States

Email: zheli@nju.edu.cn

Email: hanyangyu@nju.edu.cn

**
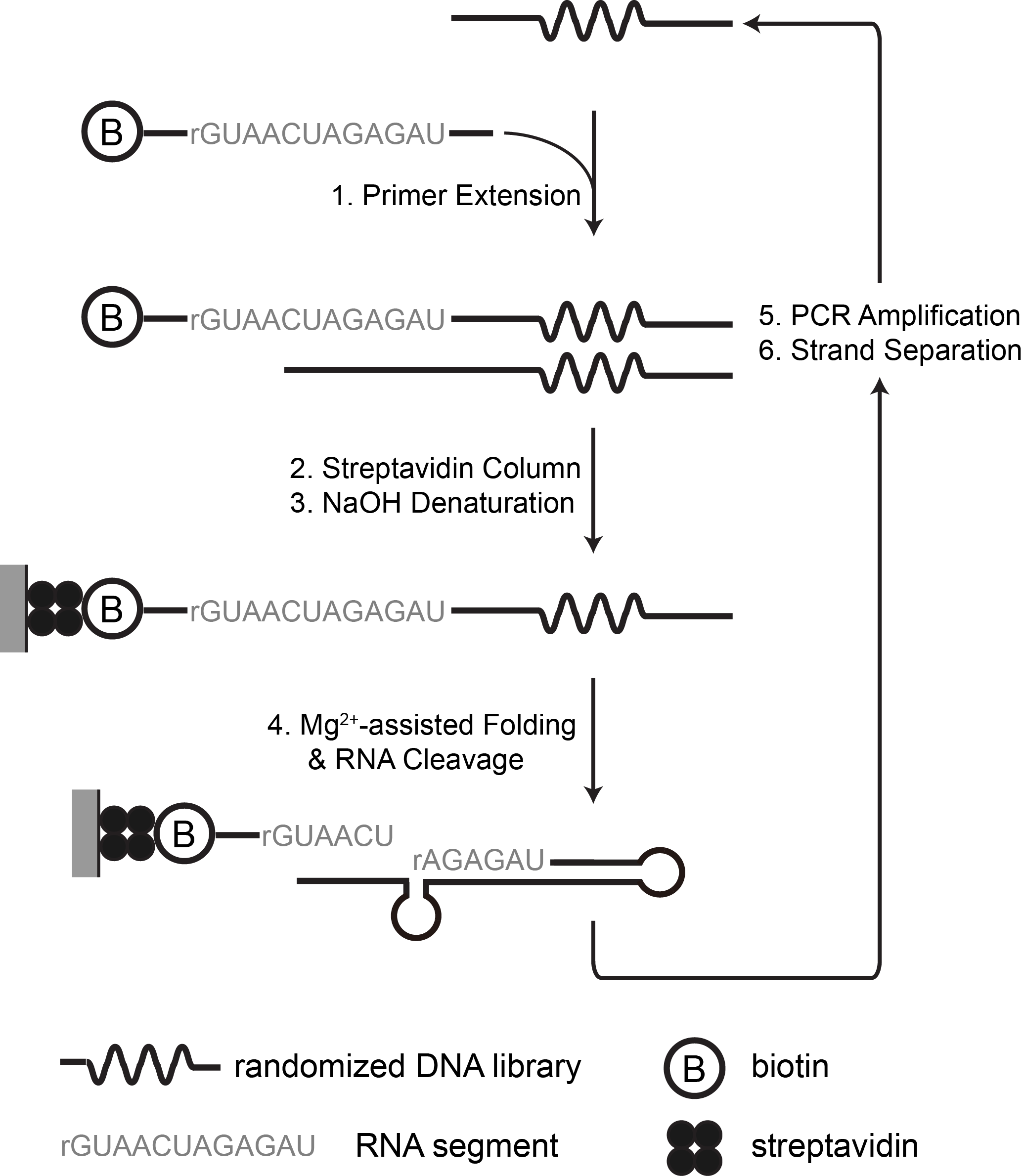
**

**Figure S1.**  In vitro selection scheme of RNA-cleaving deoxyribozymes. A DNA-RNA chimeric primer containing a segment of 12 ribonucleotides was extended to generate a DNA library with a central randomized region of 50 nucleotides. After strand separation, the library was subject to a magnesium-dependent RNA cleavage selection. Those catalytically active sequences that were able to catalyze RNA cleavage at any position with the RNA segment were collected and amplified to initiate the next round of selection.

**
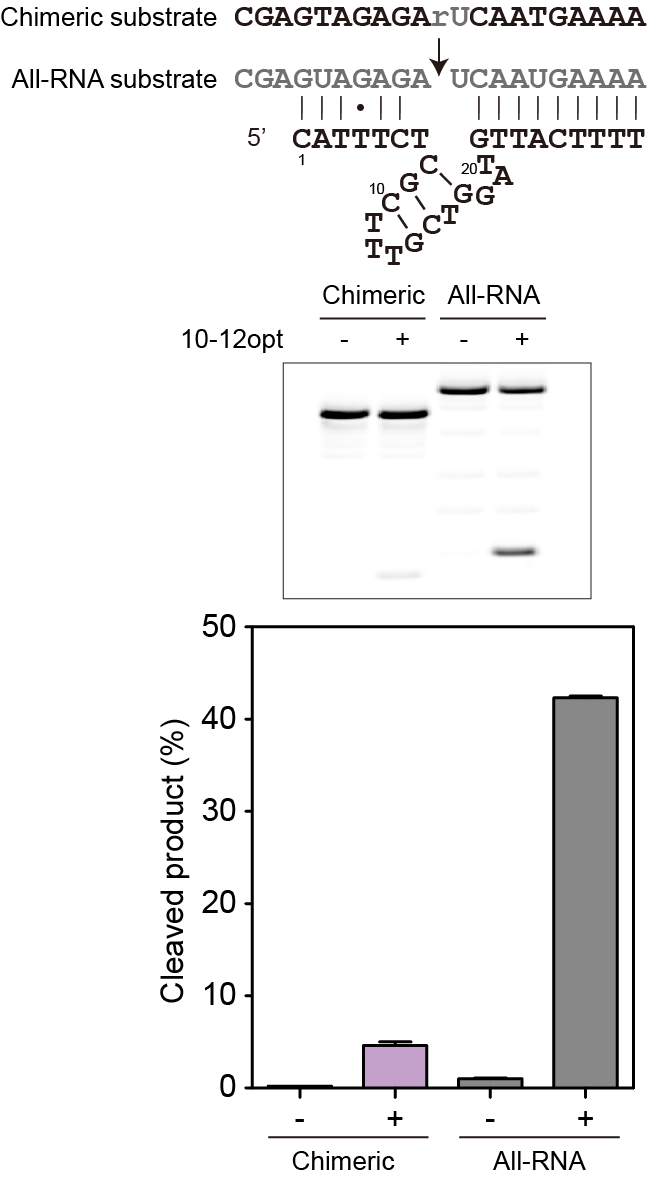
**

**Figure S2.** RNA cleavage assays of deoxyribozyme 10-12opt using DNA-RNA chimeric substrate and all-RNA substrate. The chimeric substrate contained a single ribonucleotide embedded at the potential site. The deoxyribozyme showed much reduced catalytic activity towards chimeric substrate compared with all-RNA substrate. The error bars represent the SD calculated from three independent experiments.

**
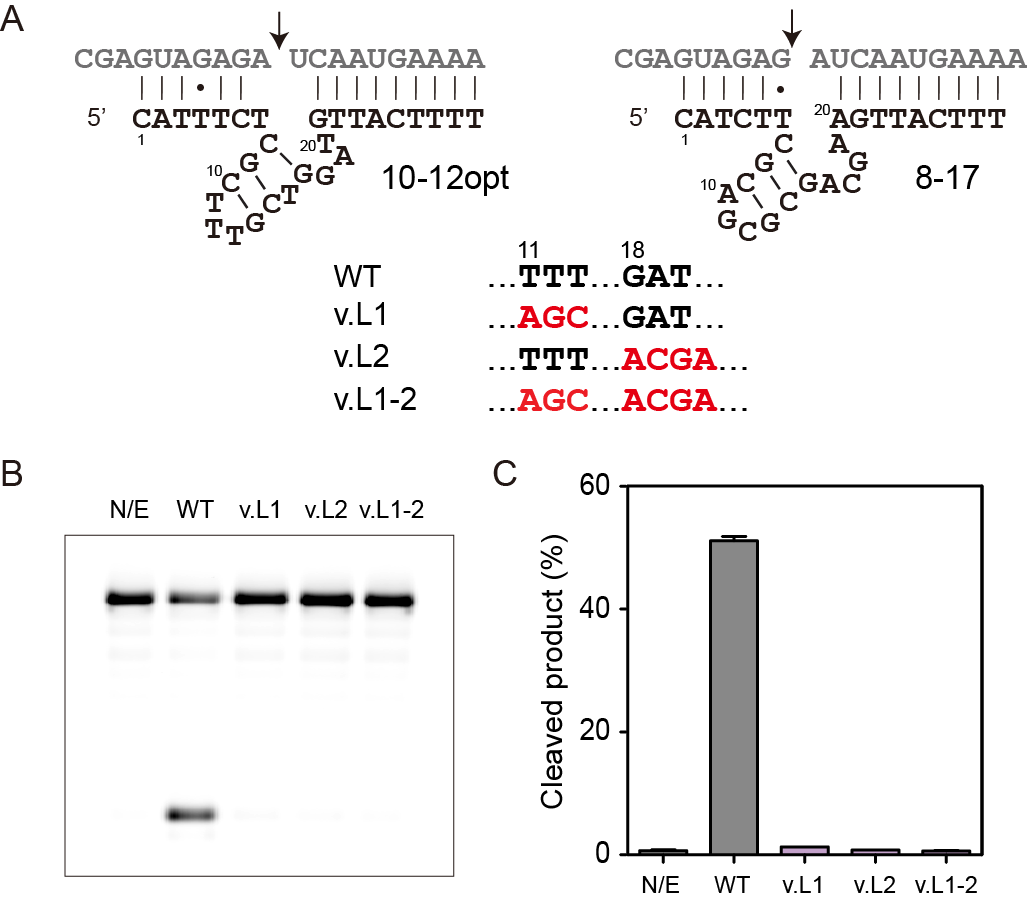
**

**Figure S3.** Substitution of two loop regions of deoxyribozyme 10-12opt with 8-17 loop sequences. (**A**) The catalytic core of 10-12opt contained two loop regions (TTT and GAT), which were replaced by the corresponding sequences found in 8-17 (shown in red), individually and simultaneously. (**B** and **C**) Deoxyribozyme variants with substituted loop sequences showed essentially no catalytic activity. The error bars represent the SD calculated from three independent experiments.

**
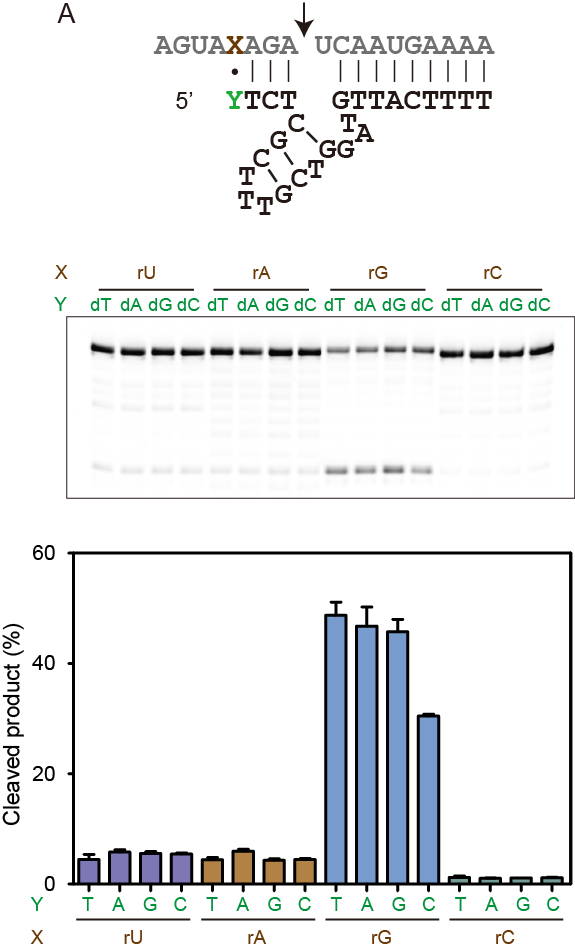
**

**Figure S4.** RNA cleavage analysis of 16 shortened deoxyribozyme-substrate combinations. Residues at position 4 of the shortened deoxyribozyme 10-12opt and at the opposite position (+4) on RNA substrate were varied. Variants of deoxyribozyme and RNA substrate were incubated in screen buffer for 3 h at 37°C, followed by denaturing PAGE analysis. Only RNA substrate with G+4 residue was preferentially cleaved by deoxyribozyme variants. Deoxyribozyme with C4 residue showed reduced catalytic activity compared with the other three variants. The error bars represent the SD calculated from three independent experiments.

**
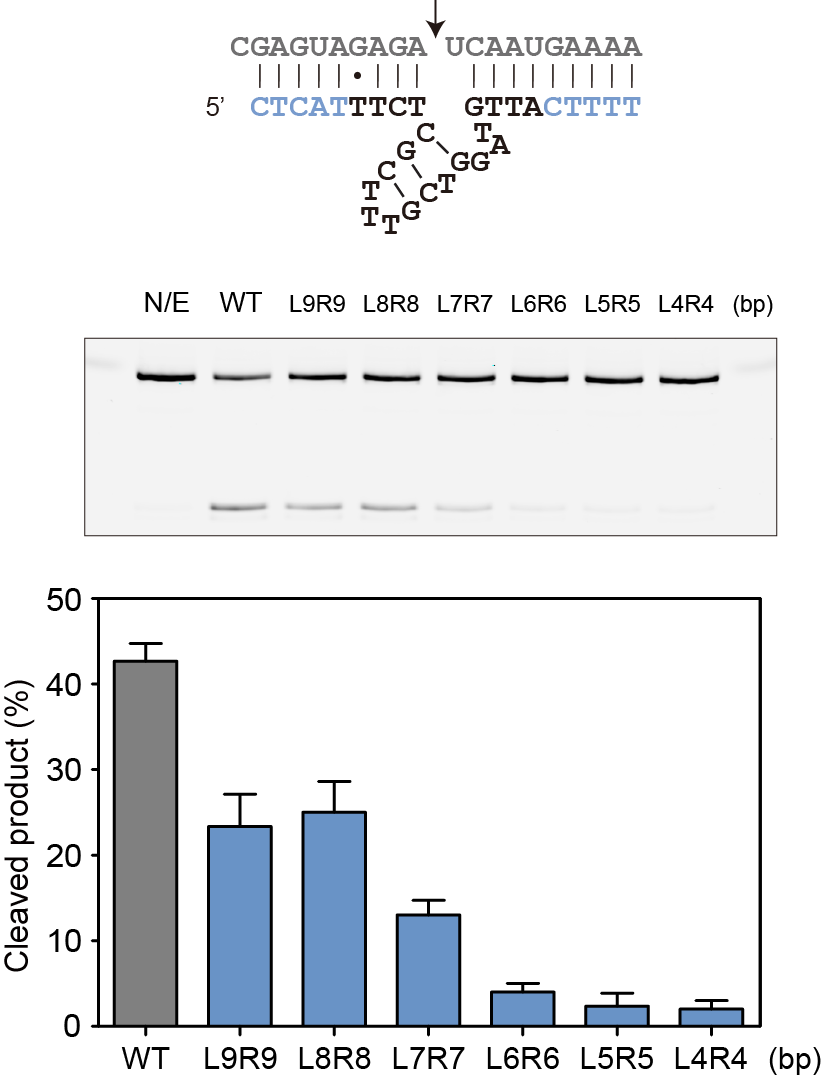
**

**Figure S5.** RNA cleavage assay using deoxyribozyme variants with simultaneously shortened left and right binding arms. Wild type (WT) deoxyribozyme 10-12opt starts with CATT and contains seven and nine nucleotides in its left and right binding arms, respectively (i.e. L7R9). The catalytic activity generally decreased with simultaneously shortened binding arms. N/E: no enzyme control. The error bars represent the SD calculated from three independent experiments.

**
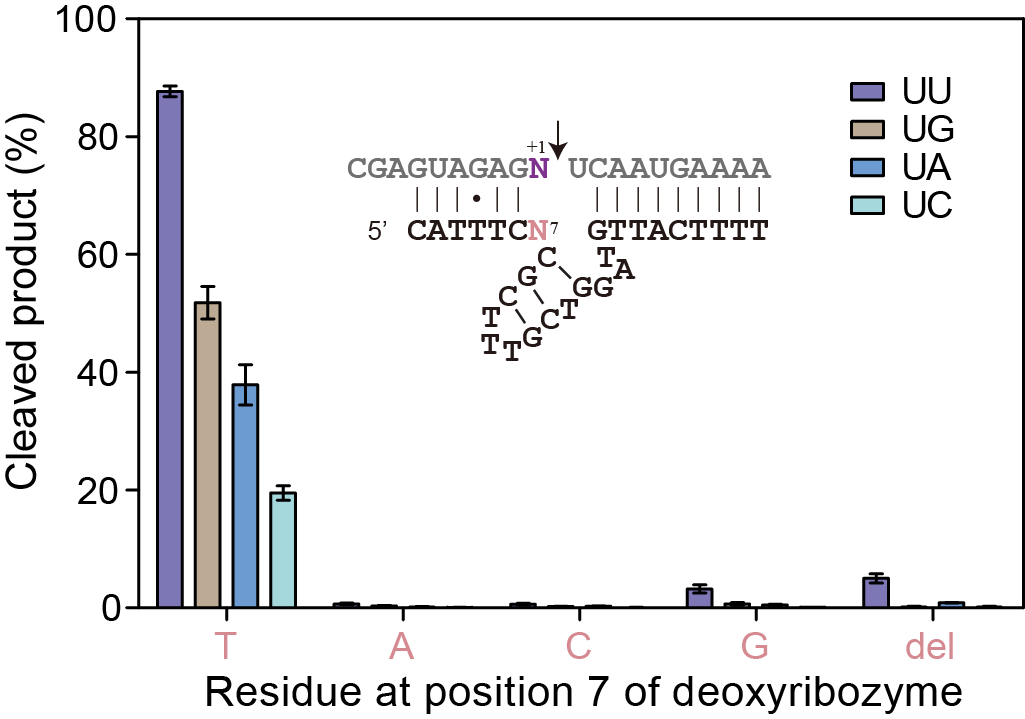
**

**Figure S6.** Deoxyribozyme 10-12opt required thymine, but not a canonical Watson-Crick base pair, at position 7 for optimal catalytic activity. Assay of a total of 20 deoxyribozyme-substrate combinations with different residues on position 7 and varied cleavage site junctions revealed that thymine at position 7 on deoxyribozyme was essential for catalysis and that deoxyribozyme catalyzed RNA cleavage with different catalytic activities in the following order: UU > UG > UA > UC. The error bars represent the SD calculated from three independent experiments.

**
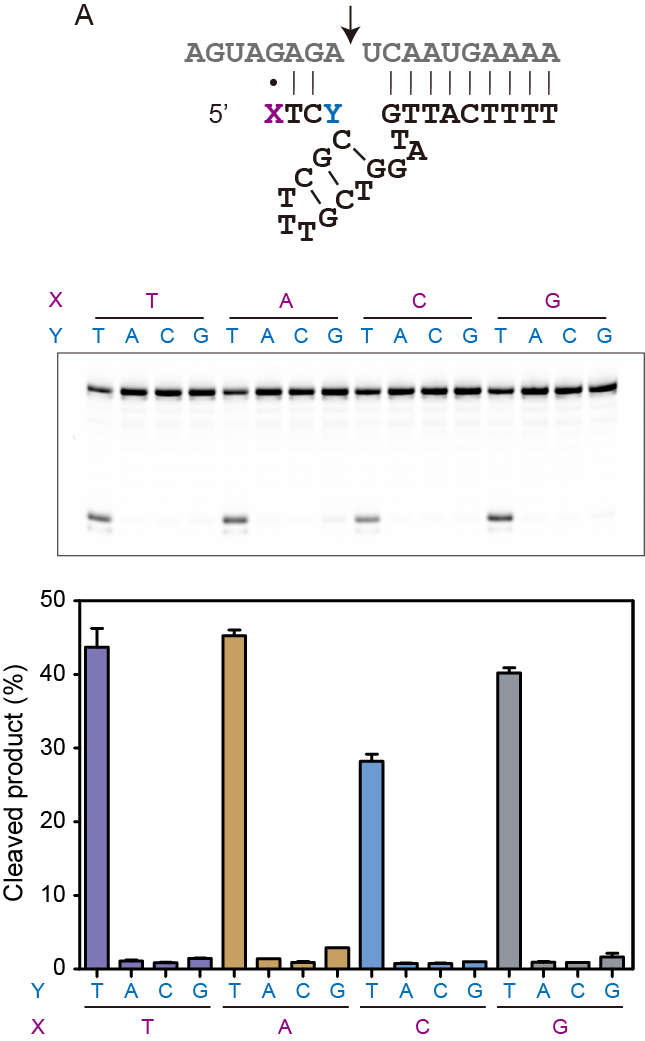
**

**Figure S7.** Mutagenesis analysis at two critical residue positions of shortened deoxyribozyme 10-12opt. The nucleotide opposite to the essential riboguanosine and the nucleotide opposite to the residue immediately downstream of cleavage site were varied. RNA cleavage assays using these 16 deoxyribozyme variants revealed that thymine at position 7 was absolutely required for optimal catalysis. Mutating T7 to any other bases completely abolished deoxyribozyme catalytic activity. Although it was tempting to assume that a base pair formed between T7 and rA+1, systematic study revealed that formation of base pair at this position was neither required nor preferred for optimal catalytic activity (Fig. 4 and S4). It was also evident that deoxyribozyme residue at position 4 had a preference for non-cytosine nucleotide. The error bars represent the SD calculated from three independent experiments.

**
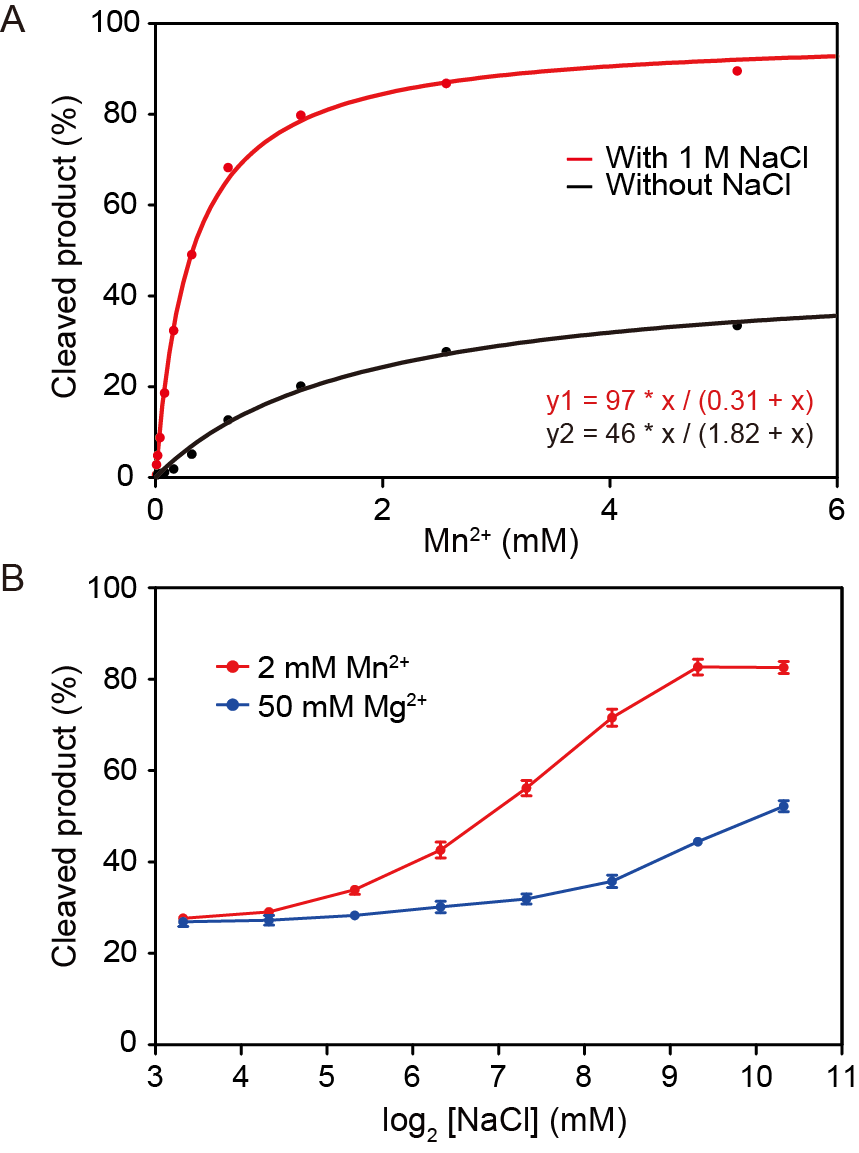
**

**Figure S8.** Effects of Mn^2+^ and NaCl on deoxyribozyme 10-12opt catalytic activity. (**A**) Mn^2+^ significantly promotes deoxyribozyme 10-12opt catalysis in the presence of 1 M NaCl in a concentration-dependent manner. (**B**) Increasing NaCl concentration in the presence of Mn^2+^ could greatly promote deoxyribozyme catalytic activity, while increasing NaCl concentration in the presence of Mg^2+^ only modestly enhanced deoxyribozyme catalyzed RNA cleavage. Deoxyribozyme-catalyzed RNA cleavage reactions were assayed in screen buffer containing different concentrations of NaCl (starting from 10 mM and incrementally increasing by 2-fold up to 1280 mM) at 37℃ for 3 hours. The error bars represent the SD calculated from three independent experiments.

**
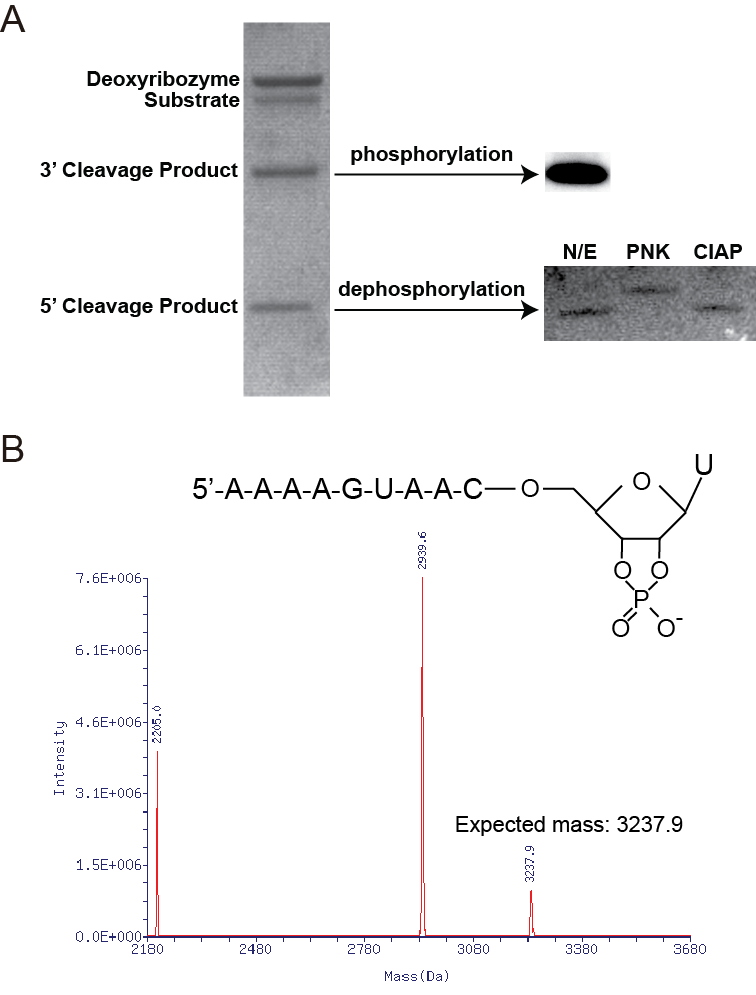
**

**Figure S9.** Identification of deoxyribozyme 10-12 cleavage products. (**A**) Overnight cleavage reaction of deoxyribozyme 10-12 was analyzed by 20% denaturing PAGE (left gel). Cleavage products were purified and processed further (right gel). The isolated 3’ cleavage product was treated with [γ-^32^P] ATP and T4 polynucleotide kinase (PNK), and was found to be successfully phosphorylated, suggesting that the 3’ cleavage product contained a free 5’ hydroxyl group. The isolated 5’ cleavage product was treated with T4 PNK and calf intestinal alkaline phosphatase (CIAP), respectively. In the absence of ATP, T4 PNK accepts substrates with a 3’-phosphate, 2’-phosphate, and 2’,3’ cyclic phosphate to produce oligonucleotides with a free 3’ terminal hydroxyl group. By contrast, CIAP accepts only 2’- and 3’-monophosphate as substrates, but does not recognize oligonucleotides bearing a 2’,3’ cyclic phosphate group. Treatment of the 5’ cleavage product with these two enzymes clearly revealed that the 5’ cleavage product contained a 2’,3’ cyclic phosphate group. N/E: no enzyme control. (**B**) Electrospray ionization spectrum of deoxyribozyme-catalyzed RNA cleavage products shows the expected mass of an upstream RNA fragment consisting of a terminal 2’,3’ cyclic phosphate group.


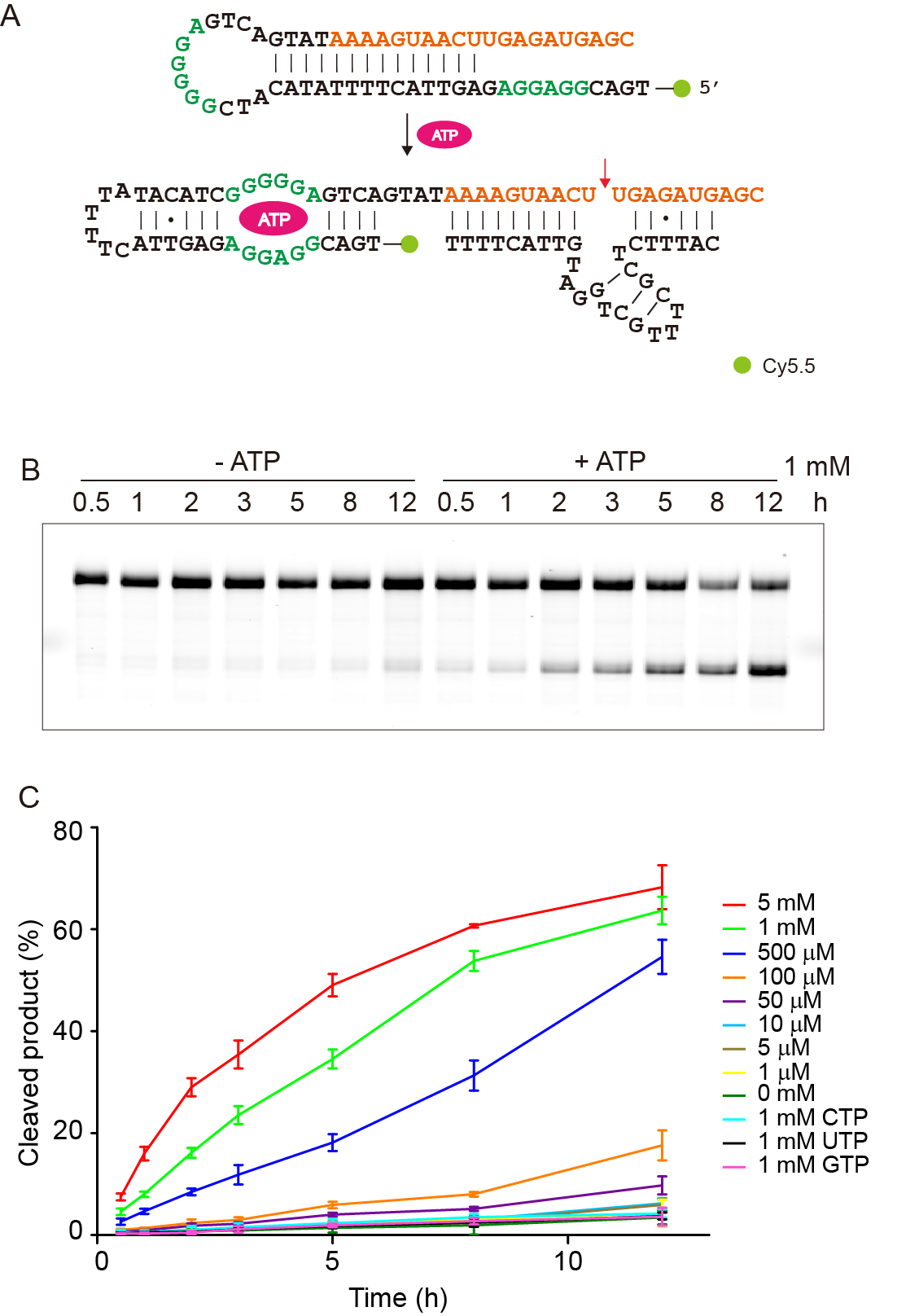


**Figure S10.** Engineering deoxyribozyme 10-12opt into an ATP sensor. (**A**) Design of a deoxyribozyme-based ATP sensor. In the absence of ATP, the RNA substrate is partially blocked by ATP aptamer sequence. ATP binding to the aptamer region induces a structural change, exposing RNA substrate susceptible to deoxyribozyme-catalyzed RNA cleavage. (**B**) Sensor performance in the absence and presence of 1 mM ATP. (**C**) Reaction kinetics of ATP sensor in response to various concentrations of ATP and other potentially interfering NTPs. The error bars represent the SD calculated from three independent experiments.

**Table S1.** Sequences of oligonucleotide library, primers, substrates and deoxyribozymes.

| Name | Sequences and modifications (5’->3’) |
| --- | --- |
| DNA library | TGTCTACACGCAAGCTTACA-N_50_-GGCTACTGCATACGAGTGTC |
| Biotinylated DNA-RNA chimeric primer | Biotin-GGAAAAA-r(GUAACUAGAGAU)-GACACTCGTATGCAGTAGCC |
| Unmodified DNA-RNA chimeric substrate | GGAAAAA-r(GUAACUAGAGAU)-GACACTCGTATGCAGTAGCC |
| PCR primer P1 | TGTCTACACGCAAGCTTACA |
| PCR primer P2 | Biotin-GACACTCGTATGCAGTAGCC |
| All-RNA substrate | r(AAAAGUAACUAGAGAUGA) |
| Cy5.5 labeled RNA substrate | Cy5.5-r(AAAAGUAACUAGAGAUGA) |
| Deoxyribozyme 8-17 | GTGTCATCTTCCGAGCCGGACGAAGTTACTTTTT |
| Deoxyribozyme 10-23 | CTTTGGTTAGGCTAGCTACAACGATTTTTCC |
| Deoxyribozyme 10-12 | TGTTTCTCGCTTTGCTGGATGTACTTTT |
| Deoxyribozyme 10-12opt | CATTTCTCGCTTTGCTGGATGTTACTTTT |
| miR-676-3p | r(CUGUCCUAAGGUUGUUGAGUU) |
| 10-12opt for miR-676-3p | TTCTCGCTTTGCTGGATCAACCTTAG |
| 8-17 for miR-676-3p | AACTCTGTCAGCGACTCGAACAACCTTAG |
| miR-3658 | r(UUUAAGAAAACACCAUGGAGAU) |
| 10-12opt for miR-3658 | TTCTCGCTTTGCTGGATTGGTGTTTT |
| 8-17 for miR-3658 | ATCTCTGTCAGCGACTCGAATGGTGTTTT |
| miR-3675-5p | r(UAUGGGGCUUCUGUAGAGAUUUC) |
| 10-12opt for miR-3675-5p | TTCTCGCTTTGCTGGATCAGAAGCCC |
| 8-17 for miR-3675-5p | AATCTCTGTCAGCGACTCGAACAGAAGCCC |
| Cy5.5 labeled ATP aptazyme | Cy5.5-TGACGGAGGAGAGTTACTTTTATACATCGG-GGGAGTCAGTAT-r(AAAAGUAACUUGAGAUGA) |

N = A:T:C:G = 1:1:1:0.5. Substrate binding arms are underlined.
